# From microaneurysm to retinal capillary macroaneurysm: Optical coherence tomographic evidence of aneurysmal evolution in diabetic retinopathy

**DOI:** 10.1016/j.ajoc.2026.102582

**Published:** 2026-04-13

**Authors:** Unnikrishnan Nair, Jay Sheth, Vineetha Vijayan, Manoj Soman

**Affiliations:** a Vitreoretinal Services, Chaithanya Eye Hospital and Research Institute, Trivandrum, India; b Chaithanya Innovation in Technology and Eyecare (Research), Trivandrum, India; c Retina Services, Shantilal Shanghvi Eye Institute, Mumbai, India

**Keywords:** Diabetic macular edema, Microaneurysm, Retinal capillary macroaneurysm, Diabetic retinopathy

## Abstract

**Purpose:**

To present a proof-of-concept case series documenting the novel in-vivo progression of retinal microaneurysms (MAs) into retinal capillary macroaneurysms (RCMAs) in diabetic retinopathy (DR) using serial optical coherence tomography (OCT).

**Observations:**

We longitudinally followed four treatment-naïve DR eyes. Over 9 to 30 months, serial OCT clearly captured the enlargement of pre-existing MAs into distinct RCMAs, characterized by a marked increase in wall reflectivity and significant morphological remodelling. This conversion consistently coincided with a notable exacerbation of localized diabetic macular edema (DME) and increased hyperreflective intraretinal dots (HRIDs), indicative of increased inflammation and leakage. The MAs originated in both inner and middle retinal layers.

**Conclusions and importance:**

Our series presents the first in vivo, multi-case demonstration of MAs dynamically enlarging into RCMAs, a clinically significant and aggressive form of diabetic microvascular disease. This transformation is consistently associated with worsening CME and increased HRIDs, marking RCMAs as a more exudative and potentially treatment-resistant subset of DME. Emerging evidence, supported by our observations, suggests that many RCMAs may arise at interconnecting vertical channels between the superficial and deep capillary plexuses, where mixed arterial-venous flow and focal pressure gradients predispose to outpouching. These findings challenge current uniform therapeutic approaches, underscoring the importance of serial-OCTs in detecting and characterizing this evolution. Early OCT-based identification could enable more tailored interventions, reducing the risk of severe vision loss. Future research should further clarify systemic and local drivers of this progression, refine OCT biomarkers, and assess whether aggressive or multimodal therapy targeting RCMAs improves patient outcomes.

## 1 Introduction

Microaneurysms (MAs) are among the earliest clinical manifestations of diabetic retinopathy (DR) and serve as important diagnostic and prognostic biomarkers.^1^ These saccular outpouchings of capillary walls result from pericyte loss, basement membrane thickening, and endothelial cell dysfunction.^1^^,^^2^ They are often associated with capillary leakage and are implicated in the pathogenesis of diabetic macular edema (DME), especially in its focal form.^1^, ^2^, ^3^ By contrast, retinal capillary macroaneurysms (RCMAs) are much larger aneurysms arising from the capillary network.^4^, ^5^ These uncommon lesions, variously defined as dilations >200 μm, have thickened hyalinized walls and often appear as solitary lobular outpouchings.^5^ Unlike classic branch retinal arterial macroaneurysms (RAMs) in large arterioles (which often cause multi-layer hemorrhage), RCMAs originate in smaller capillaries, may occur even without systemic hypertension, and are associated with chronic edema rather than acute bleeding.^5^

**Fig. 1:**
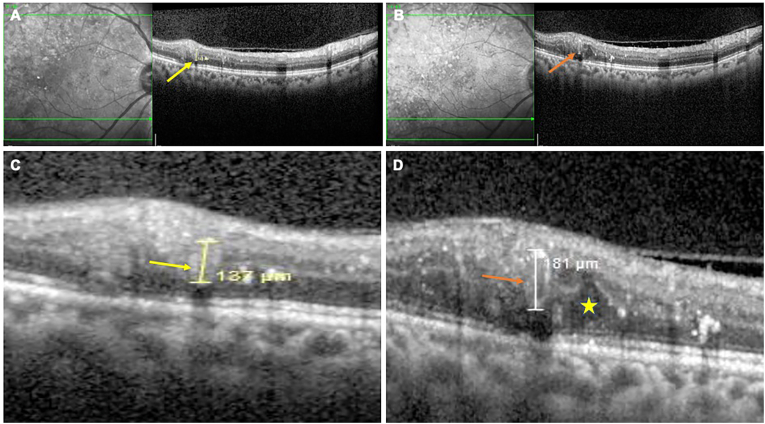
Optical coherence tomography (OCT) images demonstrating microaneurysm (MA) to retinal capillary macroaneurysm (RCMA) conversion in a 58-year-old female with moderate non-proliferative diabetic retinopathy (NPDR) and best-corrected visual acuity (BCVA) of 20/20 in both eyes. At baseline (A), a small hyperreflective dot in the inner retinal layers represented the MA (yellow arrow). On follow-up at 12 months (B), the lesion showed enlargement from 137 μm to 181 μm with transformation into a distinct RCMA (orange arrow), characterized by a prominent hyperreflective wall and variably reflective lumen, accompanied by increased cystoid spaces (yellow star) and numerous hyperreflective intraretinal dots (HRIDs). Magnified views show the baseline MA (yellow arrow; C) and the corresponding location at 12 months (D), highlighting the complete structural conversion to RCMA (orange arrow). (For interpretation of the references to colour in this figure legend, the reader is referred to the Web version of this article.)

**Fig. 2:**
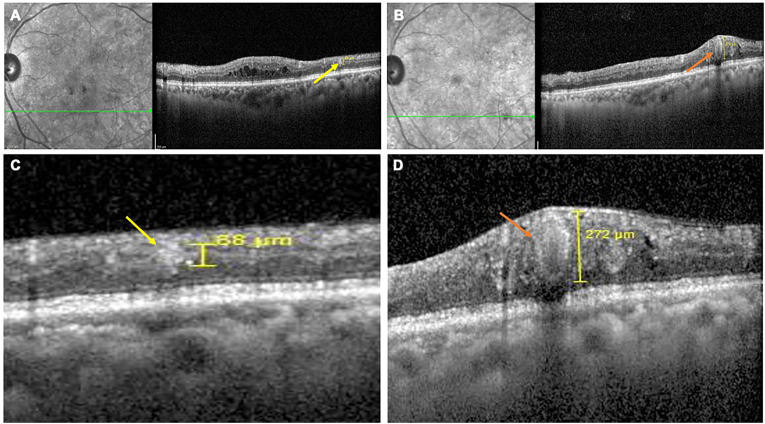
Optical coherence tomography (OCT) images showing microaneurysm (MA) to retinal capillary macroaneurysm (RCMA) conversion in a 78-year-old female with diabetes mellitus (DM) for 25 years and hypertension (HTN) for 24 years, having moderate non-proliferative diabetic retinopathy (NPDR) in both eyes with baseline best-corrected visual acuity (BCVA) of 20/20. In the left eye, the initial MA situated in the middle retinal layer (yellow arrow) gradually expanded over 30 months into an RCMA (orange arrow), with a marginal decline in BCVA to 20/30. At baseline (A), OCT shows the MA (yellow arrow), while at 30-month follow-up (B), a larger RCMA with a hyperreflective wall, heterogeneous internal reflectivity, increased cystoid spaces, and multiple hyperreflective intraretinal dots (HRIDs) is evident. Magnified views of the lesion at baseline (C) and after 30 months (D) highlight the progressive morphological transformation from MA (yellow arrow) to RCMA (orange arrow). (For interpretation of the references to colour in this figure legend, the reader is referred to the Web version of this article.)

**Fig. 3:**
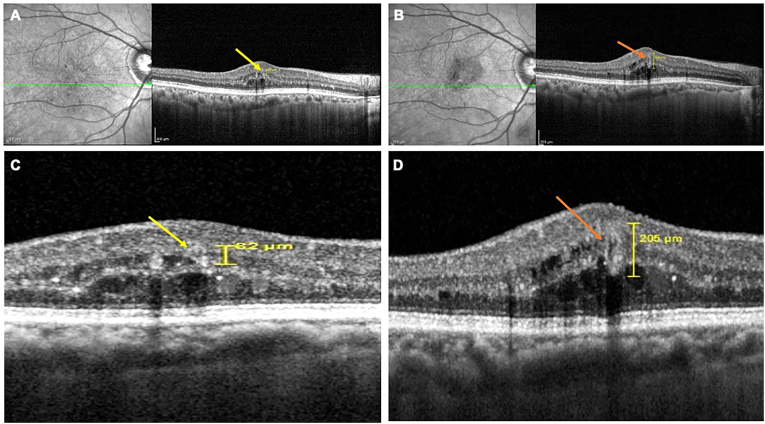
Optical coherence tomography (OCT) images illustrating microaneurysm (MA) to retinal capillary macroaneurysm (RCMA) conversion in a 72-year-old female with diabetes mellitus for 35 years, presenting with bilateral severe non-proliferative diabetic retinopathy (NPDR) and best-corrected visual acuity (BCVA) of 20/20 in both eyes. In the right eye, a rapid progression over 12 months showed the initial MA in the inner retinal layers (yellow arrow) enlarging and converting into an RCMA (orange arrow), with BCVA maintained at 20/20. Baseline OCT (A) highlights the MA (yellow arrow), while the 12-month follow-up OCT (B) demonstrates the enlarged RCMA with increased wall reflectivity, associated cystoid macular edema, and numerous hyperreflective intraretinal dots (HRIDs). Magnified images at baseline (C) and at follow-up (D) emphasize the morphological transformation and edema accompanying the RCMA development. (For interpretation of the references to colour in this figure legend, the reader is referred to the Web version of this article.)

**Fig. 4:**
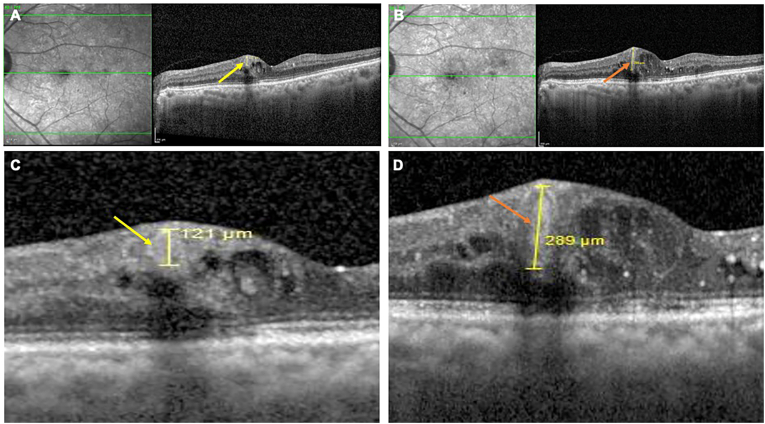
Optical coherence tomography (OCT) images demonstrating rapid microaneurysm (MA) to retinal capillary macroaneurysm (RCMA) conversion in a 63-year-old female with diabetes mellitus for 20 years and hypertension for 10 years. The right eye showed the fastest progression among cases, with MA located in the middle retinal layer (yellow arrow) transforming into an RCMA (orange arrow) within 9 months, accompanied by a marginal decline in best-corrected visual acuity (BCVA) from 20/20 to 20/30. Baseline OCT (A) depicts the initial MA (yellow arrow), while the 9-month follow-up (B) reveals an enlarged RCMA with a distinct hyperreflective wall, internal reflectivity changes, and markedly increased cystoid macular edema with numerous hyperreflective intraretinal dots (HRIDs). Magnified views at baseline (C) and follow-up (D) further illustrate the rapid morphological transformation and edema progression. (For interpretation of the references to colour in this figure legend, the reader is referred to the Web version of this article.)

The concept of an MA actually “growing into” an RCMA has not been well described. Most literature treats microaneurysms as static or sclerotic end-points of capillary damage.^1^ Spaide and Barquet^5^ first proposed “retinal capillary macroaneurysm” as a new entity after observing isolated large capillary aneurysms that expanded slowly and induced edema. Outside of case reports, it remains novel to document an ordinary MA transforming into a large macroaneurysm.

Optical coherence tomography (OCT) is uniquely suited to capture this evolution. High-resolution OCT can visualize an aneurysm's size, wall reflectivity, and adjacent fluid over time. Previous series using fluorescein or indocyanine angiography have detected RCMAs, but rarely have they been followed serially to show enlargement.^6^ For example, Karti et al.^7^ used OCT to identify a chronic “macroaneurysm” in long-standing edema, noting a large ovoid lesion with a hyperreflective wall and backshadow. Similarly, Rehmani et al.^8^ described a juxtafoveal RCMA as a spheroid inner-retinal cavity with a highly reflective wall and surrounding cystic fluid on OCT. These observations highlight OCT's ability to distinguish an expanding aneurysm from surrounding edema.

Here we report four treatment-naïve eyes with DR in which a focal MA progressively enlarged on serial OCT into a true RCMA. Each case showed concurrent worsening of localized cystoid macular edema (CME). We define the OCT criteria of MA-to-RCMA conversion (notably increased diameter and wall reflectivity) and discuss common features, mechanisms, and management implications of this novel phenomenon. RCMAs were identified per the 2025 Delphi consensus^2^ as capillary-origin lesions ≥100 μm with characteristic OCT morphology, distinct from smaller conventional MA.

## 2 Findings

### 2.1 Case 1

A 58-year-old female, who was a known diabetic since 12 years and hypertensive (HTN) since 10 years presented for diabetic eye screening. The patient had moderate non-proliferative DR (NPDR) changes in both eyes (OU) with best-corrected visual acuity (BCVA) of 20/20. The right eye demonstrated MA-to-RCMA conversion over a 12-month period with BCVA maintained at 20/20. The initial MA, located in the inner retinal layers, appeared as a small, hyperreflective dot on the OCT. Over time, follow-up imaging revealed its progressive enlargement and transformation into a distinct RCMA, characterized by a significantly larger, oval-shaped lesion with a markedly hyperreflective wall and a variably reflective lumen. This conversion was accompanied by a notable increase in localized edema, presenting as cystoid spaces with a proliferation of hyperreflective intraretinal dots (HRIDs). The increased wall reflectivity of the RCMA post-conversion was a prominent feature (Fig. 1).

### 2.2 Case 2

A 78-year old female with DM since 25 years and HTN since 24 years had bilateral moderate NPDR changes with OU BCVA of 20/20. In the left eye, the progression from MA to RCMA was observed over a longer period of 30 months with a marginal drop in BCVA to 20/30. The initial MA was situated in the middle retinal layer. Similar to Case 1, serial OCT documented the gradual expansion of this MA into an RCMA, exhibiting the characteristic hyperreflective wall and heterogeneous internal reflectivity. This morphological change was consistently associated with worsening localized macular edema, predominantly cystoid in nature, and a clear increase in HRIDs within the edematous retina (Fig. 2).

### 2.3 Case 3

A 72-year-old diabetic (since 35 years) female had bilateral severe NPDR changes and BCVA of 20/20 (OU). A rapid progression was noted in the right eye, with MA-to-RCMA conversion occurring within 12 months but BCVA maintained at 20/20. The initial MA was located in the inner retinal layers. OCT imaging captured the dynamic enlargement, culminating in the formation of an RCMA with an evident increase in wall reflectivity. Concurrently, the eye developed significant localized CME, with a marked increase in the number of HRIDs (Fig. 3).

### 2.4 Case 4

This case of a 63-year old female diabetic (20 years) and hypertensive (10 years) presented the fastest progression in the right eye, with MA-to-RCMA conversion observed in just 9 months. The BCVA reduced marginally from 20/20 at baseline to 20/30 at 9 months. The initial MA was located in the middle retinal layer. Serial OCT clearly showed the rapid transformation into an RCMA, characterized by its enlarged size, distinct hyperreflective wall, and internal reflectivity changes. This rapid conversion was tightly coupled with a pronounced increase in localized cystoid macular edema and a significant accumulation of HRIDs (Fig. 4).

## 3 Discussion

### 3.1 Overview of MA-to-RCMA transformation and OCT phenotype

In this proof-of-concept case series, we report the longitudinal transformation of MAs into RCMAs, observed over 9 to 30 months in four treatment-naïve eyes with DR. Serial OCT imaging allowed us to document this evolution in vivo, revealing a consistent pattern of gradual enlargement of pre-existing MAs, accompanied by increasing wall reflectivity, morphological remodelling, and exacerbation of localized macular edema, primarily of the cystoid variety. A critical and consistent morphological change noted after conversion was the increased reflectivity of the RCMA wall, suggesting structural alterations or accumulation of specific materials within the wall.^7^

All MA-to-RCMA conversions led to markedly worsened localized macular edema, consistently cystoid with intraretinal hyporeflective spaces. This edema was accompanied by an increase in HRIDs, which mark local inflammation or extravasated proteins,^9^, ^10^, ^11^ implying a significant inflammatory/exudative response. HRIDs often clustered around the evolving RCMAs, consistent with inflammatory exudation. The original location of the MAs varied between the inner and middle retinal layers (two cases each), indicating conversion can occur at different depths. Despite varied conversion timelines (ranging from 9 to 30 months), the final RCMA morphology (hyperreflective wall, variable lumen) and edema pattern were consistent, suggesting a distinct pathological entity. This consistent OCT signature of a hyperreflective wall with associated cystoid edema and HRIDs could serve as an OCT biomarker of aggressive, inflammation-driven DME, potentially requiring tailored therapy. The varied timelines, despite similar outcomes, underscore the complex interplay of local and systemic factors in DR progression.

### 3.2 Microvascular pathophysiology underlying aneurysmal evolution

DR pathogenesis is initiated by microvascular dysfunction, notably selective pericyte loss, basement membrane thickening and endothelial injury that disrupt inter-endothelial tight junctions and predispose to MA formation.^1^^,^^3^^,^^12^ Chronic hyperglycemia and systemic amplify oxidative stress and upregulate vascular endothelial growth factor (VEGF) signalling (including VEGF-R2),^13^ increasing capillary permeability and transmural pressure and thereby accelerating leakage.^13^

Enlargement of MA into RCMAs reflect active and sustained microvascular wall remodelling. Histology of large diabetic aneurysms (analogous to RCMAs), demonstrates elevated matrix metalloproteinase-9 (MMP-9),^5^ which degrades the basement membrane and weakens structural support.^5^^,^^14^^,^^15^ As an aneurysm expands, wall tension rises (Laplace's law:T = Pr/2h, where T is wall tension, P is transmural pressure, r is radius, and h is wall thickness),^9^ and together with MMP-9-driven degradation, creates a vicious cycle of expansion and structural fragility.^5^^,^^14^^,^^15^ This remodelling explains the observed RCMA enlargement and high wall reflectivity. In hypertensive patients, elevated transmural pressure further amplifies this wall tension.,^13^ and when combined with diabetic wall weaknesses, including pericyte loss, endothelial injury, and BM changes, this accelerates MA expansion and chronic leakage.^13^, ^14^, ^15^ Acute hyperglycemia and capillary nonperfusion compound these effects by increasing retinal hydrostatic pressure and local VEGF expression, while blood-retinal barrier disruption permits plasma proteins to extravasate and raise interstitial oncotic forces, sustaining cystoid edema.^2^^,^^5^^,^^15^^,^^16^ This interplay of mechanical and biochemical insults establishes a self-perpetuating cycle of fluid accumulation, accounting for the persistent and treatment-resistant edema associated with RCMAs.

### 3.3 Fluid dynamics, HRIDs, and the inflammatory component of RCMA-associated DME

The consistent presence and increase in HRIDs with RCMAs serve as in in vivo biomarker of local inflammation, likely activated microglia and/or extravasated lipoproteins/proteins.^9^, ^10^, ^11^ Their progressive accumulation mirros edema exacerbation, converting what may be hydrostatic leakage into a mixed hydrostatic-inflammatory phenotype. Persistent HRIDs correlate with ongoing endothelial dysfunction, barrier breakage, and refractory edema changes. These pathophysiological insights clarify why large, structurally compromised RCMAs often respond poorly to anti-VEGF monotherapy.^5^^,^^6^ VEGF blockade alone cannot reverse matrix remodelling, wall fragility, transmural stress, or sterile inflammation. Phenotype-directed therapy is therefore warranted: prolonged or intensified anti-VEGF may suppress ischemic VEGF, while corticosteroid-based interventions stabilize the barrier and mitigate inflammation. Additionally timing of therapy may also be crucial: earlier treatment of MA may prevent their enlargement and transformation into refractory RCMAs.

### 3.4 Phenotype-directed therapeutic implications and role of multimodal therapy

Focal laser photocoagulation, effective for small, isolated leaking Mas,^17^ must be applied cautiously to RCMA due to larger size, inner/middle retinal location, and partial thrombosis, which increases hemorrhage scarring risk while potentially reducing efficacy. Laser can be best used adjunctively and selectively. The convergence of structural remodelling, hydrostatic forces, VEGF-driven permeability, and local inflammation underscores the need for multimodal, individualized management strategies for RCMA-associated DME. High-resolution OCT metrics, including HRID burden, wall reflectivity, and lesion depth, enable early identification of high-risk MAs and guide proactive, tailored interventions. Our findings reinforce known DR microvascular pathology. MAs are early DR signs, indicating disease activity, and they undergo dynamic turnover, with new lesions forming as others regress.^1^^,^^3^ However, our direct serial OCT documentation of an MA enlarging into a distinct RCMA is novel. Prior reports suggested large aneurysms in DR may originate from MAs,^1^^,^^3^^,^^18^ with isolated cases of aneurysm growth,^1^^,^^3^^,^^18^ but to our knowledge no series has documented this path. This series thus provides the first multicase evidence confirming this evolutionary pathway. These findings highlight the importance of longitudinal imaging of individual MAs over time.

### 3.5 Anatomical origin of RCMAs and imaging insights from OCT/OCTA

Structurally, the superficial capillary plexus (SCP) in the inner retina is denser, while the deep capillary plexus (DCP) in the INL has lower density but supports retinal metabolism.^19^ These anatomical and hemodynamic differences influence where microaneurysms arise. We propose many RCMAs originate at vertical arteriovenous conduits linking SCP and DCP. These channels act as thoroughfares with mixed arterial/venous flow, exposed to turbulent flow, pressure gradients, and shear stress. In diabetes (pericyte loss, basement membrane thickening, endothelial damage), such forces can trigger focal wall failure and aneurysm outpouching. Depending on the site of dilation, lesions may appear superficially or more deeper on OCT, explaining variability depth. Standard OCT/OCT angiography (OCTA) slab segmentation often misclassifies these channels as SCP or DCP, masking their identity. Recognizing vertical conduits as RCMA sites reframes imaging interpretation. Histologically, such intermediate channels are known as intraretinal arteriovenous anastomoses, which our findings support. Ultimately, understanding these pathways may clarify why RCMAs behave differently. Future improvements in OCT/OCTA segmentation could better distinguish these conduits and clarify RCMA origins. OCT provides non-invasive, high-resolution, depth-resolved imaging superior to traditional fluorescein angiography for visualizing DR/DME microvasculature structures and fluid. OCTA can reveal early microvascular changes and offers quantitative analysis,^19^^,^^20^ but may miss slow-flow lesions, making structural OCT indispensable in those cases.^20, 21^ Importantly, serial OCT allows longitudinal monitoring of lesion morphology. For example, RCMAs with sluggish flow can be hard to detect on OCTA, but their structure and leakage are readily seen on OCT. This series underscores structural OCT's ability to capture dynamic changes, including wall reflectivity, lumen variability, and edema, that other modalities cannot easily discern. A notable limitation of this proof-of-concept case series is the lack of detailed systemic data (long-term glycemic control, blood pressure, and lipid profiles) that influence DR progression. Without these data, we cannot identify systemic factors contributing to MA-to-RCMA conversion or edema severity. For example, we cannot assess whether episodes of poor glycemic control or uncontrolled hypertension preceded RCMA formation. This gap limits correlating retinal findings with systemic disease and identifying risk factors for this microvascular progression. Future studies should include these parameters to better understand contributors to these outcomes.

## 4 Conclusion

This proof-of-concept case series suggests several testable hypotheses. The consistent observation of MA-to-RCMA conversion with worsening CME and increased HRIDs, points to a distinct pathological pathway in DR. Future studies should determine whether RCMAs are a specific microvascular phenotype prone to chronic leakage and inflammation, potentially requiring different therapeutic approaches than other DME forms. For example, if RCMA-associated DME responds poorly to standard VEGF therapies, this could prompt alternative treatment strategies.

The varying RCMA formation timelines (9 to 30 months) warrant investigation of factors affecting progression speed. Larger, prospective cohorts with comprehensive systemic data (glycemic control, blood pressure, inflammatory markers) are needed to identify predictors of rapid RCMA development. Longitudinal high-resolution OCT (and OCTA) studies are essential to fully characterize RCMA natural history and morphological evolution. Extended follow-up in future studies could determine whether some MAs convert even more slowly. We propose a mechanistic pathway for MA-to-RCMA evolution: diabetes causes pericyte loss and capillary wall weakness. RCMAs often arise at vertical conduits linking superficial and deep capillary plexuses, where mixed arterial-venous flow and pressure gradients causes focal outpouching. At these sites, variable pressure dynamics prevent regression. Sustained transmural pressure fluctuations and MMP-driven wall degradation drive aneurysm; Laplace's law implies rising wall tension fuels further growth. Progressive dilation increases leakage, causing cystoid edema with hyperreflective inflammatory exudates. These processes may be slowed by targeting MMP activity or reducing pressure. For example, anti-MMP agents or strict blood pressure control might slow RCMA progression. Experimentally, one could test these mechanisms using animal models or by examining human RCMA tissue (e.g., immunohistochemistry) to identify active mediators. Finally, OCT-based risk stratification could be transformative. If RCMAs or high-risk can be identified by specific OCT features, it would enable earlier, targeted interventions. Algorithms for automated detection and quantification of these lesions and biomarkers (like HRIDs) on OCT could facilitate personalized management, potentially preventing vision loss. For example, computer-based analysis could quantify MA size, wall reflectivity, and HRID count to stratify risk. Recent advances in machine learning and OCT analysis may soon make such automated risk prediction feasible in clinical practice.

## CRediT authorship contribution statement

**Unnikrishnan Nair:** Writing – review & editing, Methodology, Investigation, Conceptualization. **Jay Sheth:** Writing – review & editing, Writing – original draft, Supervision, Project administration, Formal analysis, Conceptualization. **Vineetha Vijayan:** Writing – review & editing, Formal analysis, Data curation, Conceptualization. **Manoj Soman:** Writing – review & editing, Conceptualization.

## Patient consent

Written informed consent was obtained from patients for publication of these case reports and any accompanying images.

## Claim of priority statement

After conducting a literature review on 09/09/2025 utilizing PubMed, Google Scholar, and Medline, using the key words (microaneyryms, retinal capillary macroaneyryms), we did not find any prior reports of the progression of microaneuyryms to RCMA.

## Authorship

All authors attest that they meet the current ICMJE criteria for Authorship.

## Funding

No funding or grant support.

## Declaration of competing interest

**UN, JS, VV, MS:** None.
